# Effects of drought hardening on the carbohydrate dynamics of *Quercus acutissima* seedlings under successional drought

**DOI:** 10.3389/fpls.2023.1184584

**Published:** 2023-08-25

**Authors:** Qiang Li, Xiao Liu, Xinke Sun, Mingming Zhao, Lele Liu, Ning Wang, Qun Gao, Peixian Fan, Ning Du, Hui Wang, Renqing Wang

**Affiliations:** ^1^ Institute of Ecology and Biodiversity, School of Life Sciences, Shandong University, Qingdao, China; ^2^ School of Tropical Medicine, Hainan Medical University, Haikou, China; ^3^ Qingdao Forest Ecology Research Station of National Forestry and Grassland Administration, Shandong University, Qingdao, China; ^4^ Qingdao Key Laboratory of Forest and Wetland Ecology, Shandong University, Qingdao, China; ^5^ Shandong Provincial Engineering and Technology Research Center for Vegetation Ecology, Shandong University, Qingdao, China

**Keywords:** carbon allocation, drought-trained, phenotypic plasticity, *Quercus acutissima*, stress memory, water fluctuations

## Abstract

**Introduction:**

As precipitation patterns are predicted to become increasingly erratic, the functional maintenance of warm-temperate forests constitutes a key challenge for forest managers. In this study, 2-year-old *Quercus acutissima* seedlings were selected to elucidate the mechanisms whereby they respond to soil water fluctuations and the drought hardening effects on plant carbohydrate dynamics.

**Methods:**

Seedlings were trained under different soil water conditions for 2 months: drought (D), well-watered (W), 1-month drought and then 1-month well-watered (D-W), and 1-month well-watered and then 1-month drought (W-D). The functional traits involved in water- and carbon-use strategies were explored at the end of the hardening period. Compared with seedlings in group W, seedlings in groups D, D-W, and W-D had increased potential for carbon uptake (i.e., light saturated point, maximum ribulose-1,5-bisphosphate (RuBP) saturated rate, and electron transport rate) and water uptake (i.e., fine root–to–coarse root ratio) and downregulated growth and mitochondrial respiration to decrease carbon consumption. After water fluctuation hardening, we performed a successional dry-down experiment for 1 month to detect carbohydrate dynamics and explore the acclimation caused by prior hardening.

**Results and discussion:**

Our results revealed that there were more soluble sugars allocated in the leaves and more starch allocated in the stems and roots of seedlings hardened in the D, W-D, and D-W treatments than that of seedlings hardened in the W treatment. No significant changes in total non-structural carbohydrates were found. In addition, we found near-zero (seedlings trained by D and D-W treatments) or negative (seedlings trained by W-D treatment) growth of structural biomass at the end of the dry-down experiment, which was significantly lower than that of W-hardened seedlings. This suggests that there was a shift in allocation patterns between carbon storage and growth under recurrent soil drought, which can be strengthened by drought memory. We conclude that *Q. acutissima* seedlings adjusted water- and carbon-use strategies in response to water fluctuations, whereas stress memory can enhance their overall performance in reoccurring drought. Therefore, taking advantage of stress memory is a promising management strategy in forest nurseries, and drought-trained seedlings might be more suitable for afforestation practices in sites characterized by fluctuating soil water content, considering the ongoing global climatic changes.

## 1 Introduction

Forest managers are increasingly confronted with precipitation changes, which are predicted to become more erratic, and variations in precipitation regimes will contribute to more frequent and extreme droughts (Forner et al., 2018a; Zandalinas et al., 2018). Such fluctuating events can dramatically alter forest structure and function and may cascade to affect microclimate and/or hydrology in managed forests (Barbeta et al., 2015; Forner et al., 2018b; Meakem et al., 2018). Understanding how a fluctuating climate will shape future plant communities requires a comprehensive understanding of the various drought resistance strategies that plants employ among various forest types (Reynolds et al., 2017). Warm-temperate regions cannot evade these global episodes, as there is always a spring drought, and, afterward, precipitation is abundant and rapid in summer, leading to soil water fluctuations (Luan et al., 2011; Corlett, 2016). Maintaining ecosystem services in these warm-temperate forests requires managing them as complex adaptive systems (Webster et al., 2018).

Coping with seasonal drought, trees in warm-temperate regions have developed suites of strategies to respond to variations in precipitation patterns (Cavender-Bares et al., 2004; Liu et al., 2019; Salmon et al., 2019). A well-adapted tree species may show either a more plastic response or a more stable performance to environmental variations (Valladares et al., 2002; Gratani, 2014). Phenotypic plasticity allows trees to resist water deficit along a range between two opposing strategies: drought avoidance vs. drought tolerance. Some studies have shown that, within species, individuals can adopt both avoidance and tolerance strategies. It appears that some plants can even switch between avoidance and tolerance strategies based on environmental conditions and developmental stages (Martínez-Vilalta and Garcia-Forner, 2017). Therefore, it seems reasonable to define avoidance/tolerance along a continuum rather than as a dichotomy. From a resource economics perspective, drought avoidance traits are expected to be associated with resource acquisition, whereas drought tolerance traits are associated with resource conservation (Reich et al., 1997; Wright et al., 2004). Consequently, increased drought avoidance is expected to be linked to increased resource capture, whereas increased drought tolerance is hypothesized to be associated with reduced growth potential (Reich, 2014; Ramírez-Valiente and Cavender-Bares, 2017). Such stable functional traits may allow plants to mitigate variations under environmental conditions, and plastic responses can help plants occupy a wider range in climate space. Thus, a better understanding of trait modification would be helpful for forest management in improving seedlings’ performance after transplanting (Oliet et al., 2013).

The abruptness of extreme episodes gives plants little time for the acclimation process, but a “stress memory” can lead to a faster response and better performance when they are faced with recurrent stress (Walter et al., 2013). A drought memory might augment the transcript levels of drought-responsive genes and/or induce hormonal changes to help provide drought adaptation (Bruce et al., 2007; Diego et al., 2015; Yan et al., 2017; Khan et al., 2021). Drought hardening during the seedling stage is an effective method because younger plants are more malleable and more able to survive under stressful conditions (Zhang et al., 2018). Some evidence has indicated the importance of drought hardening in woody seedlings. For example, hardening treatment might enhance drought tolerance by physiological adjustment (in *Quercus ilex* seedlings; Villar-Salvador et al., 2004), by osmoregulation and biochemical pathways (in *Jatropha curcas* seedlings; Yang et al., 2015), or by morphological changes and aquaporin expression (in *Cryptomeria japonica* seedlings; Saiki et al., 2020). In addition, seedling hardening can improve the survival rate and growth adaptability after transplanting (Huang et al., 2013). Thus, drought hardening can be applied to woody seedlings as a methodology for forest management to improve their field performance.

Non-structural carbohydrates (NSCs; principally, soluble sugars and starch) are involved in multiple plant physiological functions. The two components of NSCs have different functions under stress (Richardson et al., 2015; Jiang et al., 2020). The immediate functions of soluble sugars can directly support plant production, whereas starch serves as an important energy store for future use and as a source of soluble sugars. In general, the distinction between immediate needs versus future storage is important for the role of NSCs in coping with external stress (He et al., 2020). NSC conversion dynamics and their distribution patterns in different organs represent an indicator of the status of a tree’s carbon balance and are critically important for woody plants to tolerate biotic and abiotic stress (Richardson et al., 2015; He et al., 2020). In addition, the total carbohydrate pool size per plant reflects the “fueling status” and is the critical determinant of plant growth and survival (Porter and Kitajima, 2007; Richardson et al., 2013). Thus, it is necessary to study carbon balance strategies with the aim of gaining a more comprehensive understanding of plant responses to drought.

Considering their ecological dominance, remarkable diversity, and growing ecological data resources (Cavender-Bares, 2019), oak species (genus *Quercus*) are used as a model clade for the study of plant ecophysiology, plant–insect interactions, and ecosystem processes in the persistence of populations of long-lived organisms, which tend to face more variable environments and fluctuating selection over their life spans than short-lived organisms (Cavender-Bares, 2016). Differences in the distribution of tree species on a local scale suggest that they must develop adaptations that allow them to successfully establish and survive under a given level of resources (Edwards et al., 2014; Aguilar-Romero et al., 2017; Steckel et al., 2020). *Quercus acutissima* is native to China and dominant in warm-temperate forests, and it has a winter-deciduous leaf habit (Yuan et al., 2013; Zhang et al., 2013). It has significant ecological importance, such as soil water conservation and carbon sequestration. *Quercus acutissima* is characterized by a strong episodic growth habit; thus, it can be more easily affected by seasonal dynamics and threatened by water fluctuations related to climate change (Mazis et al., 2020; Molina-Valero et al., 2021). Thus, from forestry and ecological standpoints, characterizing how *Q. acutissima* seedlings’ response traits are integrated can provide information and guidance for practical management in warm-temperate oak forests.

The use of appropriate management strategies to enhance the adaptive capacity of warm-temperate forests has been increasingly argued by scientists (Luan et al., 2011; Corlett, 2016; Webster et al., 2018). For example, mixing and thinning can help in growth performance but hinder drought resistance and resilience (Sohn et al., 2016; Jacob et al., 2021). Site-specific forest management during the early plantation stages is scarce but critical for adapting to local conditions and maintaining its future sustainability (Lecomte et al., 2022). The aim of this study was to evaluate the effect of *Q. acutissima*’s acclimation generated from soil water fluctuations on subsequent drought exposure. For this purpose, we focused on the relative importance of phenotypic plasticity and response stability and excavated key functional traits potentially involved in this fluctuation event. We analyzed multiple traits at the whole-plant level, which have been broadly documented to be involved in drought responses and have been reported to be under natural selection in dry environments (Ramírez-Valiente et al., 2010). Seedlings were sampled from four different soil water treatments. Our specific objectives were (i) to assess seedlings’ ecophysiological responses to soil water fluctuations (i.e., phenotypic plasticity vs. response stability) and (ii) to demonstrate the presence of acclimation generated from prior hardening by estimating the effect of this acclimation on seedlings’ carbohydrate allocation dynamics under recurrent successional drought. The potential to merge “stress memory” theory into restoration activities was also discussed.

## 2 Materials and methods

### 2.1 Study site and seedlings growth

This study was conducted at the Fanggan Research Station of Shandong University in the Central Mountainous Region of Shandong Province, China (36°26′N, 117°27′E). The local climate is a warm-temperate monsoon climate with an average annual precipitation of 600–800 mm (mostly from June to August) (Yuan et al., 2013). Acorns were collected at the end of autumn under individual *Q. acutissima* trees growing on a hill near the research station. The seeds were germinated in humectant sand in the greenhouse, and the germinants were planted in 50-cell plug trays for 1 month. Then, they were transplanted to 12.5-L plastic pots (32 cm in depth and 29 cm in diameter) containing 8 kg of growth substrate. The plant growth substrate was a mixture of air-dried sandy loam and commercial organic soil in proportions of 4:1 by volume. All seedlings were grown in the greenhouse and watered and fertilized biweekly with 20:20:20 N:P:K fertilizer solution (0.5 g L^−1^) in their first year. Two-year-old seedlings were used in the subsequent greenhouse experiment, where the polyethylene plastic roof protected seedlings from natural precipitation, and open ends provided sufficient ventilation for the inside temperature to remain close to the ambient environment.

Two experiments were carried out during the growth season of the second year. The temperature and relative humidity were logged every 5 min (HOBO data loggers, Onset, Bourne, MA, USA) in the greenhouse. The mean temperature was 32°C in the daytime and 21°C at night, and the mean relative humidity was 60% in the daytime and 95% at night throughout the course of the experiments.

### 2.2 Water fluctuation experiment

We conducted a pilot experiment to determine the water dosages of drought and well-watered treatments. Seedlings in the drought treatment were kept at ~15% of field water capacity (where the leaves started to wilt according to pre-experiment), and, in the well-watered treatment, they were kept at ~70% for good growth. Both the drought and well-watered treatments were applied by daily weight and irrigation after sunset. The water fluctuation experiment lasted for 2 months, comprising four groups: (1) drought treatment for 2 months (D), (2) well-watered treatment for 2 months (W), (3) drought treatment for 1 month and then well-watered treatment for 1 month (D-W), and (4) well-watered treatment for 1 month and then drought treatment for 1 month (W-D). A complete block design was applied, in which 80 2-year-old seedlings were randomly arranged into four groups (*n* = 20) and randomly distributed in the greenhouse space. The initial heights and basal diameters were recorded (36.5 cm in mean height and 5.5 mm in mean basal diameter; no significant difference among the four groups). At the end of the fluctuation experiment, we randomly selected 10 *Q. acutissima* seedlings from each group for harvesting, and the remaining 10 individuals were used for the dry-down experiment.

At the end of the water fluctuation experiment, we first measured the seedlings’ heights and basal diameters to determine growth during this phase. We then established photosynthetic curves in response to light and CO_2_ with a portable infrared gas analyzer (Li-6800, Li-Cor, Lincoln, NE, USA). The measurements were taken in a fully developed sun-exposed leaf per seedling between 9:00 and 11:00. For the light-response curves, the ambient concentration of CO_2_ was fixed at 400 μmol mol -1 (ppm), and the photosynthetic photon flux density (PPFD) decreased slowly from 2,000 to 0 μmol m^−2^ s^−1^ (2,000, 1,800, 1,500, 1,200, 1,000, 800, 500, 300, 200, 100, 80, 50, 20, and 0 μmol m^−2^ s^−1^). The maximum photosynthetic rate (*A*_m_), light saturated point (LSP), and light compensation point (LCP) were calculated from the curves fitted by a nonrectangular hyperbola equation (Thornley, 1976). In the CO_2_ response curves, the PPFD was fixed at 1,500 μmol m^−2^ s^−1^, and the ambient CO_2_ concentration started at 400 μmol mol^−1^, decreased slowly to 50 μmol mol^−1^ and stabilized at 400 μmol mol^−1^ and then increased to 1,500 μmol mol^−1^ (400, 300, 200, 100, 50, 400, 400, 600, 1,000, and 1,500 μmol mol^−1^) following the work of Stinziano et al. (2019). The mitochondrial respiration rate (*R*_m_), maximum rate of carboxylation (*V*_cmax_), and electronic transport (*J*_max_) were calculated from the curves fitted by the Farquhar–von Caemmerer–Berry model (Farquhar et al., 1980).

After the curves were established, leaf samples were collected to determine leaf morphology [leaf thickness, specific leaf area (SLA), and vein density (VD)], leaf biochemistry (chlorophyll content), and leaf water status (leaf water content and leaf water potential). Stem samples were collected to determine stem morphology (bark thickness and wood density) and stem hydraulic traits (stem water potential and stem hydraulic conductivity).

For each seedling, we collected 10 mature sun-exposed leaves with no visible damage to measure the fresh mass, leaf area (using ImageJ version 1.51j8; National Institutes of Health, Bethesda, MD, USA), and leaf thickness. Leaf thickness was determined as the average thickness at the top, middle, and bottom of the leaf using an electronic digital micrometer. The samples for leaf vein measurement were initially preserved in formalin–acetic acid–alcohol solution (FAA; 37% formaldehyde, glacial acetic acid, 95% ethanol in proportions of 5:5:90 by volume). One leaf was sampled from each of 10 individuals per treatment, and three subsamples of 1 cm × 1 cm were cut from the top, middle, and bottom portions and preserved in FAA before measurement. Leaf subsamples were cleaned for 1 week in a 10% NaOH aqueous solution, stained with 0.1% safranin for 15 min, rinsed in distilled water, and mounted on transparent sheets. The samples were photographed at ×10 magnification with a light microscope (CX31RTSF, Olympus, Tokyo, Japan). Vein lengths were determined from five digital images via AJ-VERT software (X64, AOR Industrial CO., LTD, Shenzhen, China), and the values for VD were recorded as vein length per unit area (McElwain et al., 2016). Five leaf disks (6 mm in diameter) of each seedling were taken to measure chlorophyll content according to Inskeep and Bloom (1985). In brief, chlorophyll was extracted with an 80% acetone solution until the residue was colorless. The acetone extract was then filtered and fixed at a constant volume, and the absorbance was measured at 663 nm (*A*_663_) and 645 nm (*A*_645_). The contents of chlorophyll a (Chl_a_), chlorophyll b (Chl_b_), and total chlorophyll (Chl_t_) were calculated as follows:


$${\text{Chl}}_{a}=12.71A_{663}-2.59A_{645}$$

$${\text{Chl}}_{b}=22.88A_{645}-4.67A_{663}$$

$${\text{Chl}}_{t}=20.29A_{645}+8.04A_{663}$$

A 3-cm-long stem piece above the root collar was cut, and the bark (including all phloem) was peeled off for bark thickness and woody density calculation. Bark thickness was determined by the difference between the stem diameter with bark and the stem diameter without bark. The wood volume was determined by the water displacement method (Rungwattana and Hietz, 2017) and was calculated as the ratio of dry mass to volume. Stem-specific hydraulic conductivity (*K*s) was measured in fragments 10 cm in length using a self-made hydraulic apparatus. Briefly, the segments were connected to a degassed and filtered KCl solution (20 mmol L^−1^) with hydrostatic pressure generated gravitationally. The downstream end of the segment was connected to a graduated pipette, and the time required for the meniscus in the pipette to cross a certain number of consecutive graduation marks was recorded. The hydraulic conductivity was calculated as the ratio of the flow rate through the segment to the pressure gradient, and the stem-specific hydraulic conductivity (*K*s) was calculated as the ratio of the hydraulic conductivity to the stem cross-sectional area. Midday leaf water potential (Ψ_leaf_) was measured between 12:00 and 13:00 with a Scholander pressure chamber (1505D-EXP, PMS Instrument, Albany, OR, USA). At the same time, stem water potential (Ψ_stem_) was estimated by measuring the water potential in a leaf covered with aluminum foil for 1 h to allow the leaf water potential to equilibrate with the stem xylem water potential, and the water potential difference between the stem and leaf (Ψ_stem-leaf_) was calculated by Ψ_stem_ − Ψ_leaf_.

After the leaf samples and stem samples were collected, 10 seedlings from each group were harvested. The roots were gently cleaned of soil particles using flowing tap water and then absorbed using paper towels. Light-colored, succulent, and unsuberized rootlets were chosen for respiration measurement within 5–10 min to minimize the effect of excision on respiratory activity (Rodríguez-Calcerrada et al., 2017). Root samples were laid flat side-by-side in the chamber, and the root respiration rate was recorded until CO_2_ release was constant. It is possible that the *in vivo R*_root_ was higher because of the microenvironment changes. To minimize the effect of excision on respiratory activity, stable readings were taken in 2 min. All samples were placed in the sample chamber (6 cm^2^) for gas exchange measurements, so *R*_root_ was expressed per root area. The maximum root length was also measured with a ruler. To measure dry biomass, all plant parts were placed in a drying oven at 60°C for 72 h, and leaves, stems, fine roots (< 2 mm in diameter), and coarse roots (> 2 mm in diameter) were weighed separately. The whole-plant dry mass (DM_plant_) was defined as the sum of the leaf biomass, stem biomass, fine root biomass, and coarse root biomass. The root-to-shoot biomass ratio (*R*/*S*) and the fine root–to–coarse root biomass ratio (Fr/Cr) were calculated.

After measuring the tissue dry biomass, all samples were ground to a fine homogeneous powder with a ball mill, and the powder was then used for the analysis of NSC concentration, using modified protocols described by Hansen and Møller (1975).

When the assessment of all traits was completed, a relative distance plasticity index (RDPI) ranging from 0 (no plasticity) to 1 (maximal plasticity) was obtained for each trait, according to Valladares et al. (2006):


$$\text{RDPI}=\;\sum^{\;}\left(d_{ij\to i^{\prime }j^{\prime }}/\left(x_{ij}+x_{i^{\prime }j^{\prime }}\right)\right)/n,$$

where x*_ij_* is the trait value of a given individual *j* (*j* = 1, …, 10) subjected to water treatment *i* (*i* = 1, …, 4); the distance among trait value d*_ij_*_→_ *_i’j′_* for all pairs of individuals for which *i* is different from *i′* (the two individuals were grown under different water treatments) is the absolute value of the difference x*_ij_* − x*_i’j′_* when *i* ≠ *i′*, and the relative distances are defined as ${\text{d}}_{ij\to i^{\prime }j^{\prime }}/\left(x_{ij}+x_{i'j'}\right)$ ; and *n* is the total number of distances. We defined drought avoidance traits that were associated with the resource acquisition strategy, whereas drought tolerance traits were associated with the resource conservation strategy, according to Ramírez-Valiente and Cavender-Bares (2017). In addition to calculating the RDPI for each trait, we calculated the mean RDPI of avoidance traits and tolerance traits, as well as overall traits.

### 2.3 Dry-down experiment

To detect the effect of water fluctuation hardening on seedlings’ carbon dynamics, we randomly selected 10 *Q. acutissima* seedlings from each treatment for a successional dry-down experiment, which was applied by withholding water for 1 month. At the end of the fluctuation experiment, we randomly selected 10 *Q. acutissima* seedlings in each treatment for a successional dry-down experiment for 1 month to detect the seedlings’ carbon dynamics. At the end of the dry-down phase, all seedlings were harvested to detect NSC consumption during a 1-month dry-down. We measured the soluble sugar and starch concentrations in the leaf, stem, and root tissues, and we calculated the ratio of soluble sugar to starch concentrations (SS/St) to illustrate NSC conversion dynamics. The NSC mass was calculated as NSC concentration × biomass, and the structural carbohydrate (SC) mass was calculated by subtracting the NSC mass from the biomass (Weber et al., 2018). To detect the structural growth through the dry-down phase, relative SC mass changes were calculated as the differences between the SC mass at the beginning and the end of the whole dry-down phase; values > 0 indicated a net increase, whereas values< 0 indicated a net decrease.

### 2.4 Calculations and statistics

Light response curves and CO_2_ response curves were fitted with function *nlsLM* from the *minpack.lm* package and function *fitacis* from the *plantecophys* package, respectively, in R Statistical Software v.3.3.1 (R Development Core Team, 2016). To test the effect of the treatments on trait values, a one-way analysis of variance (ANOVA) was performed. All assumptions of ANOVA were met, and least significant difference (LSD) multiple comparisons were performed when the treatment effects were significant. The tests were considered significant when *P<* 0.05. Differences among the RDPI groups (avoidance trait RDPI, tolerance trait RDPI, and overall RDPI) were tested for significance with non-parametric Friedman’s ANOVA. ANOVA tests were conducted using the SPSS 23.0 software package (SPSS Inc., Illinois, USA).

## 3 Results

### 3.1 Effects of water fluctuation treatment

A one-way ANOVA indicated significant differences in both avoidance and tolerance traits among treatments (**Table 1**). Compared to well-watered seedlings, drought generated a significant reduction in the values of *J*_max_, *R*_m_, Ψ_leaf_, Ψ_stem_, RL, *R*/*S*, DM_Cr_, and DM_root_ and a significant increase in Ψ_stem-leaf_ and *K*_s_ (**Table 1**). The W-D water fluctuation treatment generated the lowest values of *J*_max_, Ψ_leaf_, and Ψ_stem_ but the highest values of Ψ_stem-leaf_ and BT (**Table 1**). The D-W water fluctuation treatment generated the highest values of LSP, *K*_s_, Ψ_leaf_, Ψ_stem_, and Ψ_stem-leaf_ but the lowest values of BT, SS_root_, and St_leaf_ (**Table 1**).

**Table 1:** Mean values of avoidance and tolerance traits among drought (D), well-watered (W), and water fluctuation (W-D and D-W) treatments.

Variable	Soil water treatment				RDPI
	W	D	W-D	D-W	
Avoidance traits					
*A*_max_ (μmol m^−2^ s^−1^)	12.87 ± 3.22	11.5 ± 2.69	13.91 ± 1.86	17.98 ± 1.56	0.34
LSP (μmol CO_2_ m^−2^ s^−1^)	1,083.58 ± 200.7^a^	1,201.04 ± 179.58^ab^	1,159.08 ± 110.76^ab^	1,695.38 ± 259.65^b^	0.27
LCP (μmol CO_2_ m^−2^ s^−1^)	204.87 ± 52.2	210.43 ± 53.1	207.21 ± 22.89	221.54 ± 36.51	0.38
*V*_cmax_ (μmol m^−2^ s^−1^)	43.4 ± 6.03^ab^	28.22 ± 5.69^a^	26.49 ± 3.34^a^	52.89 ± 7.56^b^	0.32
*J*_max_ (μmol m^−2^ s^−1^)	76.67 ± 9.99^b^	45.41 ± 10.53^a^	38.79 ± 6.67^a^	92.92 ± 11.5^b^	0.36
Chl_a_ (mg mm^−2^)	5.04 ± 0.46	4.54 ± 0.35	5.12 ± 0.43	4.98 ± 0.44	0.14
Chl_b_ (mg mm^−2^)	2.73 ± 0.26	2.57 ± 0.18	2.82 ± 0.23	3.17 ± 0.18	0.14
Chl_t_ (mg mm^−2^)	7.76 ± 0.72	7.11 ± 0.52	7.94 ± 0.65	7.11 ± 0.91	0.14
SLA (cm^2^ g^−1^)	2,311.01 ± 232.74	2,783.11 ± 302.62	2,400.19 ± 284.44	2,227.18 ± 608.99	0.21
Ψ_leaf_ (MPa)	−12.19 ± 0.68^b^	−14.36 ± 1.06^c^	−14.54 ± 0.55^c^	−6.69 ± 0.94^a^	0.18
Ψ_stem_ (MPa)	−10.62 ± 0.51^b^	−12.59 ± 0.9^bc^	−12.88 ± 0.58^c^	−5.79 ± 0.89^a^	0.19
Ψ_stem-leaf_ (MPa)	1.57 ± 0.3^a^	1.77 ± 0.3^b^	1.66 ± 0.29^b^	1.94 ± 0.36^c^	0.35
VD (mm mm^−2^)	6,327.21 ± 188.13^ab^	5,895.2 ± 234.41^ab^	6,005.69 ± 192.3^b^	3,749.94 ± 830.44^a^	0.07
*K*_s_ (kg m^−1^ s^−1^ MPa^−1^)	1.78 ± 0.45^a^	2.06 ± 0.59^b^	1.41 ± 0.41^ab^	2.09 ± 0.46^ab^	0.47
RL (cm)	34 ± 1.57^b^	30.05 ± 0.99^a^	35.19 ± 1.26^b^	33.16 ± 1.38^ab^	0.07
DM_Fr_ (g)	4.4 ± 0.97	2.9 ± 0.4	3.65 ± 0.58	3.77 ± 0.61	0.29
Fr/Cr	0.21 ± 0.02^ab^	0.21 ± 0.02^a^	0.2 ± 0.01^ab^	0.24 ± 0.03^b^	0.19
*R*/*S*	1.8 ± 0.26^b^	1.33 ± 0.09^a^	1.41 ± 0.09^ab^	1.58 ± 0.2^ab^	0.19
SS_root_ (mg g^−1^)	15.16 ± 0.88^b^	16.33 ± 1.08^b^	14.16 ± 1.58^b^	10.65 ± 0.64^a^	0.17
SS_stem_ (mg g^−1^)	9.18 ± 0.61^ab^	8.39 ± 0.53^ab^	13.29 ± 3.49^b^	8.05 ± 0.54^a^	0.18
SS_leaf_ (mg g^−1^)	16.42 ± 1.47	16.79 ± 2	16.72 ± 1.69	18.48 ± 1.15	0.15
Tolerance traits					
*R*_m_ (μmol m^−2^ s^−1^)	2.7 ± 0.14^b^	2.08 ± 0.2^a^	1.72 ± 0.22^a^	2.74 ± 0.12^b^	0.19
*R*_root_ (μmol m^−2^ s^−1^)	4.2 ± 0.26	3.4 ± 0.15	3.8 ± 0.42	3.75 ± 0.19	0.12
*H* (cm)	36.05 ± 2.48	33.47 ± 2.97	34.17 ± 3.6	41.72 ± 2.43	0.15
BD (mm)	5.29 ± 0.3	5.22 ± 0.52	5.78 ± 0.45	5.07 ± 0.21	0.13
LT (mm)	0.22 ± 0.01	0.22 ± 0.01	0.21 ± 0.01	0.22 ± 0.01	0.05
DM_leaf_ (g)	9.01 ± 1.36	8.38 ± 0.82	9.36 ± 1.13	7.62 ± 0.61	0.21
DM_stem_ (g)	5.28 ± 0.9	4.42 ± 0.59	6.47 ± 1.17	4.93 ± 0.65	0.26
SD (g mm^−3^)	0.71 ± 0.04	0.73 ± 0.04	0.75 ± 0.03	0.76 ± 0.03	0.08
BT (mm)	10.58 ± 0.65^b^	12.71 ± 0.81^bc^	13.56 ± 0.57^c^	7.53 ± 0.88^a^	0.19
DM_Cr_ (g)	21.11 ± 4.04^b^	13.59 ± 1.01^a^	17.68 ± 2.21^ab^	14.73 ± 1.22^ab^	0.22
DM_root_ (g)	25.51 ± 4.9^b^	16.49 ± 1.25^a^	21.33 ± 2.78^ab^	18.5 ± 1.75^ab^	0.22
DM_shoot_ (g)	14.49 ± 2.11	12.9 ± 1.32	15.83 ± 2.25	12.55 ± 1.14	0.22
DM_plant_ (g)	40 ± 6.69	29.39 ± 2.41	37.16 ± 4.85	31.05 ± 2.26	0.20
St_root_ (mg g^−1^)	39.04 ± 3.9	45.61 ± 4.45	44.02 ± 2.91	45.95 ± 3.81	0.16
NSC_root_ (mg g^−1^)	54.2 ± 3.77	61.94 ± 4.51	58.18 ± 2.87	56.61 ± 4.06	0.23
St_stem_ (mg g^−1^)	34.77 ± 1.71	31.71 ± 1.91	31.27 ± 3.27	35 ± 1.25	0.12
NSC_stem_ (mg g^−1^)	43.95 ± 1.73	40.1 ± 1.71	44.56 ± 3.08	43.06 ± 1.39	0.15
St_leaf_ (mg g^−1^)	17.55 ± 0.94^b^	15.75 ± 0.9^b^	14.54 ± 1.12^ab^	12.59 ± 1.24^a^	0.15
NSC_leaf_ (mg g^−1^)	33.97 ± 1.79	32.55 ± 2.32	31.26 ± 1.47	31.07 ± 1.79	0.10
LWC	0.93 ± 0.02	0.88 ± 0.02	0.92 ± 0.01	1.63 ± 0.41	0.06

A_max_, maximum photosynthetic rate; BD, basal diameter; BT, bark thickness; Chl, concentration of chlorophyll; DM, dry mass; Fr/Cr, fine root–to–coarse root biomass ratio; H, height; J_max_, electron transport rate driving RuBP regeneration; K_s_, stem hydraulic conductivity; LCP, light compensation point; LSP, light saturated point; LT, leaf thickness; LWC, leaf water content; NSC, non-structural carbohydrate concentration; R_m_, mitochondrial respiration rate; RL, maximum root length; R_root_, root respiration rate; R/S, root-to-shoot biomass ratio; SD, stem density; SLA, specific leaf area; SS, soluble sugar concentration; St, starch concentration; V_cmax_, maximum RuBP saturated rate of carboxylation; VD, vein density; Ψ water potential.

The mean ± SE (n = 10) is shown in the table. Letters are shown when significant differences between treatments were detected (P< 0.05). The relative distance plasticity indices (RDPI) of each trait are shown.

### 3.2 RDPI values: avoidance traits versus tolerance traits

**Figure 1:**
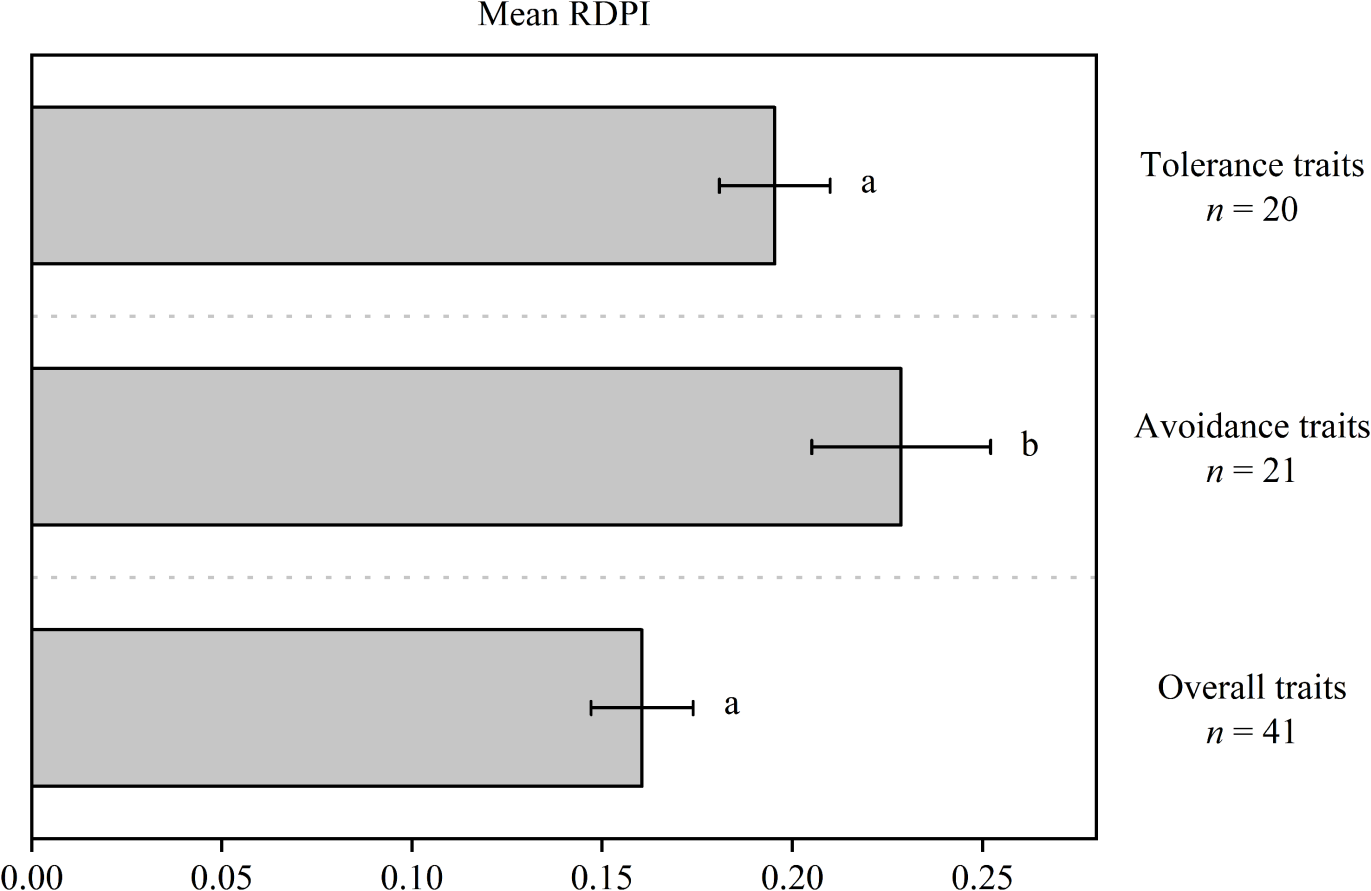
Mean relative distance plasticity index (RDPI) of avoidance, tolerance, and overall traits. Different letters indicate significant differences (*P<* 0.05).

Trait plasticity in response to soil water fluctuations was highly variable, from low trait plasticity (e.g., RDPI_LT_ = 0.05) to high trait plasticity (e.g., RDPI_LCP_ = 0.38) (**Table 1**). Non-parametric ANOVA indicated significantly higher RDPI values for avoidance traits (0.23) than for tolerance (0.16) and overall traits (0.19) (**Figure 1**).

### 3.3 Final NSCs after a successional dry-down

**Figure 2:**
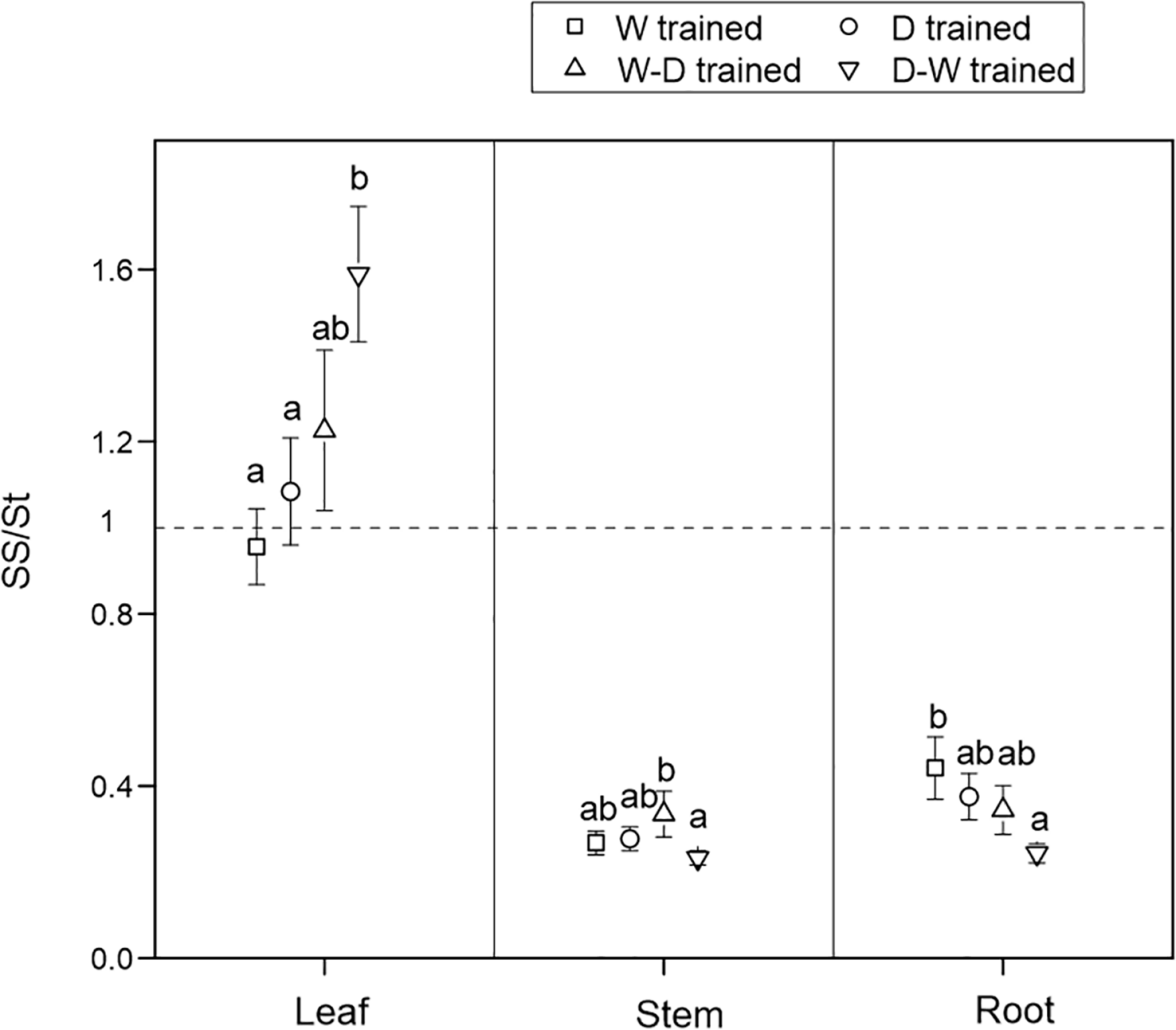
Ratio of soluble sugar to starch concentrations (SS/St) among tissues after a 1-month successional dry-down. Data are mean ± SE; *n* = 10. Different letters indicate significant differences (*P<* 0.05) within organs.

At the final harvest, after a 1-month successional dry-down treatment, SS/St varied in all tissues among the four groups. There were more soluble sugars than starch in the leaves but more starch in the stems and roots. Compared with the W seedlings, the D-W–hardened seedlings showed the highest values of SS/St in leaves but the lowest values of SS/St in the roots (**Figure 2**).

**Figure 3:**
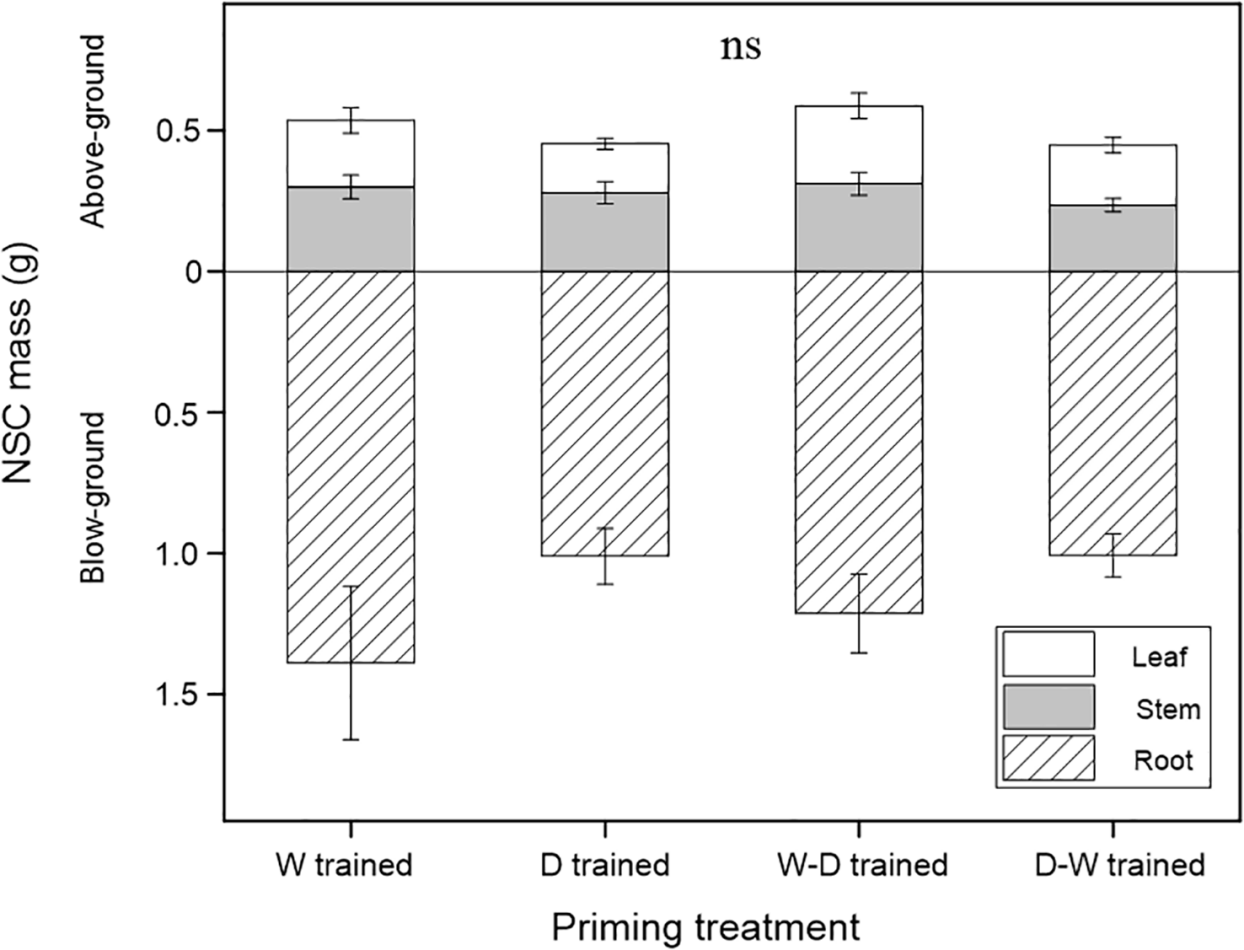
NSC mass among the well-watered group (W), drought group (D), and water fluctuation groups (W-D and D-W) after a 1-month successional dry-down period. Data are mean ± SE; *n* = 10. Non-significant differences are shown as ns.

At the end of the experiment, NSC mass differences among the four groups were not significant either for the tissue (i.e., leaves, stems, and roots) level or for the whole-plant level (**Figure 3**).

**Figure 4:**
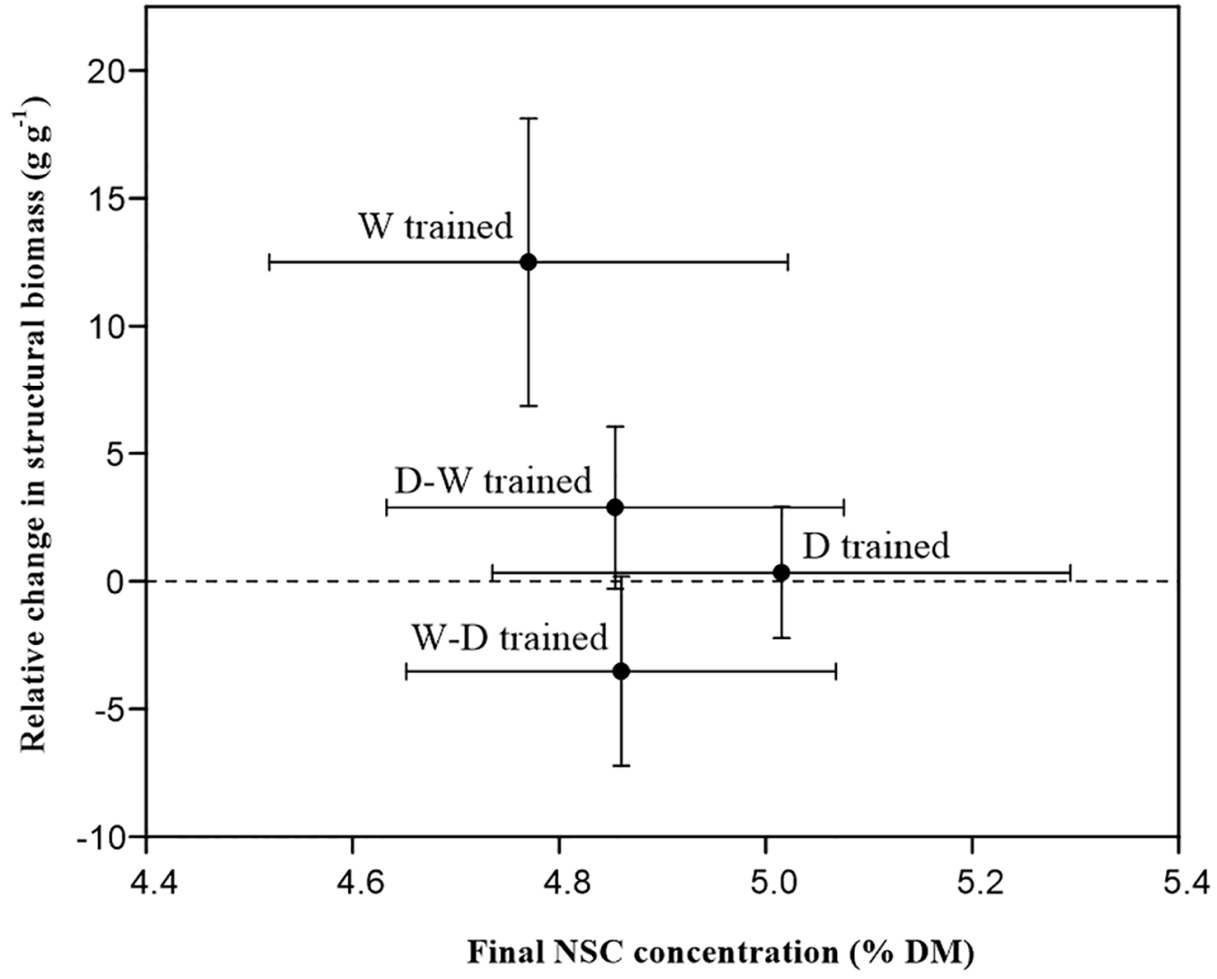
Relative structural biomass changes in different treatments related to their NSC concentrations after a 1-month successional dry-down period. Data are mean ± SE; *n* = 10.

The potential shift in allocation patterns between structural growth and NSC storage was investigated. At the end of the dry-down experiment, there was a near-zero (seedlings trained by D and D-W treatments) and negative (seedlings trained by W-D treatment) growth of structural biomass, whereas the final NSC concentrations were maintained at ~5% of dry mass (**Figure 4**).

## 4 Discussion

### 4.1 Trait responses under soil water fluctuations

Consistent with the expected developmental flexibility of woody species in response to environmental variations, *Q. acutissima* seedlings showed a wide range of trait-specific variations, that is, both significant changes and stable responses, from leaf traits and hydraulics to growth and biomass allocation patterns. This could be the result of adaptation to seasonal dryness in warm-temperate zones. The association of species strategies with the environmental conditions of species’ native ranges is crucial for forecasting species performance under varying rainfall conditions, especially in the context of the changing climate, which is predicted to become more extreme and variable (Balaguer et al., 2001; Koehler et al., 2012; Kaproth and Cavender-Bares, 2016).

For most measured leaf traits, the overall variation arose in physiological traits and biomass allocation, rather than in chemical or anatomical traits. Specifically, CO_2_ assimilation and usage traits (i.e., LSP, *V*_cmax_, *J*_max_, and *R*_m_) increased, whereas light interception traits (e.g., chlorophyll content and SLA) and *A*_max_ were not significantly affected (**Table 1**). Thus, we infer that *Q. acutissima* seedlings take the strategy of maintaining light interception (unaffected chlorophyll contents and SLA), upregulating light use efficiency (increased LSP, *V*_cmax_, and *J*_max_), and depressing respiration consumption (decreased *R*_m_) to maintain carbon assimilation and production of newly produced carbohydrates (Thomas, 2009). In addition, the overextension of the leaf area might result in increased tension in the xylem and further impair conductivity (Trugman et al., 2018; Zhang et al., 2019). Such potential hydraulic risk was avoided by *Q. acutissima* seedlings; that is, no significant SLA changes were found among soil water fluctuation treatments (**Table 1**).

The biomass allocation patterns among plant tissues could characterize the strategy of proportional investment in water-absorbing, water-conducting, and water-transpiring, and it is generally considered the dominant process by which plants adjust their hydraulic systems in response to drought (Choat et al., 2018). In this study, seedlings invested proportionately more in fine roots than coarse roots when suffering drought (**Table 1**), which supports the functional equilibrium hypothesis—plants tend to maximize their surface area for the acquisition of the most limiting resource (Reynolds et al., 2017), in this case, water. Unexpectedly, seedlings invested proportionately less in belowground biomass (the addition of fine root and coarse root biomass) than in aboveground biomass, but the root length was not significantly influenced by drought (**Table 1**). This may be a result of the limited capacity to spend on coarse root construction and root elongation. Heredia-Guerrero et al. (2014) proposed that oak seedlings had a conservative pattern of root biomass allocation in response to nutrient availability; thus, we speculate that this conservative pattern also occurs in response to water availability. Thus, we should remember the caution of choosing key traits when classifying the “conservative” response or the “acquisitive” response to stress.

### 4.2 Mean plasticity: avoidance traits versus tolerance traits under soil water fluctuations

The correlation between water availability and the species’ stress resistance (tolerance, avoidance, or both) is a strong indication of species adaptability to their native environment (Kaproth and Cavender-Bares, 2016). *Quercus acutissima*, native to China with a history of seasonal drought, was defined as an isohydric response species in our previous work (Li et al., 2019) and displayed a higher avoidance ability than tolerance under fluctuating soil water conditions in the present study. In general, the plasticity of avoidance traits ranged from 0.07 to 0.38 and that of tolerance traits ranged from 0.05 to 0.26 (**Table 1**), and the mean level of the plasticity of avoidance traits was significantly higher than tolerance traits (**Figure 1**). A smaller tolerance trait plasticity relative to avoidance trait plasticity may be because the alteration of tolerance traits (traits associated with resource conservation here) requires a relative greater cost than avoidance traits (traits associated with resource acquisition here) (Westerband et al., 2019). Overall, it is reasonable to note from the data here that *Q. acutissima* seedlings behaved as drought-avoiding species.

Nevertheless, *Q. acutissima* displayed a high plasticity for a few traits under particular water availabilities, mainly for biomass accumulation and NSC dynamics, providing some insights into plastic strategies that may enhance plant performance under stress. Carbon storage may promote the ability of plants to perform better in the future, such as in stress survival and post-stress recovery (Galvez et al., 2011; Fallon and Cavender-Bares, 2018). NSC dynamics allow seedlings to cover respiratory demands and osmoprotection to maintain membrane integrity (Sanz-Pérez et al., 2009; Sun et al., 2018), which has been proven to be a primary process that increases drought tolerance after hardening (Farooq et al., 2009). Thus, it seems that high plasticity in carbon dynamics is important for better stress resistance.

### 4.3 NSC dynamics after recurrent drought

After 1 month of successional drought, soluble sugars and starch conversion patterns varied in all tissues among the four groups (**Figure 2**), but there were no significant differences in NSC consumption (**Figure 3**). We found that all seedlings in the four groups had proportionally more movable sugars in leaves but more stored NSC in the form of starch in stems and roots, and this conversion pattern was more evident in D-W seedlings (**Figure 2**). This could reflect the conversion patterns of sugars (“fast” pool, which is readily transported and metabolically active) to starch (“slow” pool, which is immobile and metabolically inert) (Richardson et al., 2013). Fast cycling is conserved in easily replaceable tissue (i.e., leaf), and slow cycling is conserved in persistent tissues (i.e., stem and root), reflecting the carbon conversion dynamics in investment strategies among source–sink tissues (Fatichi et al., 2018). NSC storage (i.e., starch) is critically important for woody plants because these reserves are sufficient to buffer carbon deficits and enable sessile, long-lived organisms to tolerate biotic and abiotic stress, including drought (Klein and Hoch, 2015; Richardson et al., 2015). If environmental changes are rapid enough that plants experience novel conditions, then evolutionary responses may be more important than immediate responses (Franks et al., 2013). Accordingly, we also propose that carbon starvation rarely occurs in *Q. acutissima* seedlings, even during the successional drought process, which might be due to the downregulation of carbon consumption (Richardson et al., 2013).

### 4.4 Allocation patterns between carbon storage and growth after recurrent drought

Growth and survival are tightly correlated with fitness, and growth responses are thought to be more sensitive than other physiological traits (Sáenz-Romero et al., 2017). Acclimation to environmental stress may involve investment in resistance mechanisms that enhance survivorship under stress, potentially at the cost of limiting growth (Kaproth and Cavender-Bares, 2016). There is ongoing debate regarding the possibility of carbon allocation patterns between growth and storage, where trees invest preferentially in carbon storage at the expense of growth under unfavorable conditions (Wikberg and Ögren, 2007; Sanz-Pérez et al., 2009). Our results suggest that growth rates are modified to ensure a target NSC concentration (about 5% dry mass) when soil water is limited (**Figure 4**). As evidence, the growth of structural biomass almost stopped in the D-W–hardened and D-hardened seedlings, and a negative relative change was found in the W-D–hardened group during the recurrent successional drought period (**Figure 4**). Such a threshold suggests that a shift in allocation patterns between growth and storage occurred among drought-hardened seedlings in this experiment and points toward a mechanism allowing seedlings to “sense” their total reserve pool size (Blackman et al., 2019; Weber et al., 2019). Similar mechanisms have also been demonstrated for another *Quercus* species (Weber et al., 2018) and a *Casuarina* species (Blackman et al., 2019). Thus, we can infer that *Q. acutissima* seedlings take the strategy of keeping whole-plant NSC concentrations relatively stable under successional drought stress by decreasing carbon consumption to allocate more carbon for storage, especially in drought-hardened seedlings, which is linked to the capacity to resist prolonged stress conditions (Rodríguez-Calcerrada et al., 2017; Xie et al., 2018). In addition, fast growth is always associated with a high sensitivity to drought, as the hydraulic transport capacity is too low to support rapid water consumption (Wikberg and Ögren, 2007). Thus, we address a pressing need to determine the fitness effects and ecological relevance of physiological responses (Medeiros and Danielson, 2018). The trade-off between stress resistance and growth persistence reminds us that introducing faster-growing species might not necessarily be successful. A species used in the afforestation activities should be evaluated from both growth ability and stress-resistance performance, considering that climate change is more unpredictable.

### 4.5 Acclimations as a drought training effect

We found more evident NSC conversion and a shift in allocation patterns between growth and storage in drought-hardened seedlings (especially for seedlings from the D-W group) when faced with a recurrent successional drought (see Section 4.3). In our study, drought-hardened seedlings, especially in the D-W group, showed improved performance in physiological and morphological traits. Specifically, there was increased photochemical efficiency and physiological vitality and a better hydraulic status after 2 months of training (**Table 1**). In addition, *Q*. *acutissima* seedlings had a strong ability to maintain the whole-plant NSC pool (i.e., NSC mass; see **Figure 3**) at an approximately stable level, even under unfavorable conditions. The size of a tree’s NSC pool reflects its carbon “fueling status” (Richardson et al., 2013) and is critical for post-stress recovery. It is reasonable that *Q. acutissima*, as a long-lived species with the ability to resprout, may have to allocate NSC to long-term storage and keep the NSC pool stable at the expense of other sinks to ensure survival, which can be thought of as an adaptive strategy to local climate with a history of seasonal drought. Hence, previous exposure to stress can provide the benefit of enhanced protection without the costs associated with constitutive expression of related genes when suffering this kind of stress, which can even be carried forward to the next generation (Bruce et al., 2007). Our study supports the potential application of seedlings’ drought training, and this pre-existing adaptive capacity can enhance seedlings’ drought resistance after transplant. In particular, exposure to drought and well-watered hardening are more effective. It is reasonable to call for further investigations of the hardening effect concerning specific mechanisms and more species and stress types.

## 5 Conclusions

Our study provides a new approach to classifying drought-avoiding or drought-tolerant species via the relative values of the RDPI. Along with this classification, we demonstrated that *Q*. *acutissima* seedlings, as drought-avoiding species, respond to environmental conditions through both performance stability and phenotypic plasticity. Among these response traits, biomass allocation and NSC dynamics mostly reflect drought resistance, that is, keeping the NSC pool stable and preferring storage allocation under drought. Thus, it is reasonable to highlight the need to consider multiple trade-offs and the importance of finding key traits reflecting species’ response strategy precisely. Hence, we recommend that restoration ecologists drought-train seedlings in the nursery prior to transplant, especially where seasonal droughts are common.

## Data availability statement

The raw data supporting the conclusions of this article will be made available by the authors, without undue reservation.

## Author contributions

QL, QG, PF, ND, HW, and RW proposed the study and designed the experiment. QL conducted field and laboratory experiments and analyzed the data. ND, HW, and RW secured funding. XL, XS, and MZ helped with laboratory experiments and data analysis. LL and NW helped with data analysis. QL wrote the manuscript, which was intensively edited by all authors. All authors contributed to the article and approved the submitted version.
